# Editorial

**Published:** 1851-04

**Authors:** 


### THE MEDICAL EXAMINER.

PHILADELPHIA, APRIL, 1851.

### REGISTRATION OF BIRTHS.

Our attention has recently been drawn to the fact, that the Board of Health of this City, by a resolution, have notified the practitioners of midwifery within their jurisdiction to furnish complete returns of the number of births, during the years 1848, '49, and '50. It is deeply to be regretted, that there should have occurred any neglect in collecting these statistics for the last three years, as it shows an apparent dereliction of duty somewhere. It is the more to be lamented, as there is at all times a serious difficulty in procuring such records, and, since a register of births occurring in a large city from year to year, constitute a prominent feature in the tables of vital statistics. In consequence of this hiatus, the Board of Health have failed to complete and publish their annual statement of Births and Deaths for the years above named.

Another source of regret is, that when the missing statistics shall be collected, and especially those embracing the births for 1849 and '50, during and following the prevalence of Epidemic Cholera, they will be, at the best, so imperfect, that only very meagre results can be obtained from them, more particularly as to the effect of the epidemic, and its depressing influences upon the vital energies, in their relation to the reproductive organs.

We have ever hesitated to believe that the fault in obtaining a correct and connected statement of births, lay at the doors of the practitioners, or, that they have not viewed the science of vital statistics of sufficient import to demand their careful study and appreciation, or that they can fail to remember, that these statistics have for their object, the discovery of those laws of the economy of our nature, by which births, marriages, disease and death, under every variety of circumstances, are regulated.

But we apprehend, from the action of the Board, that the failure must have been attributed, in some degree at least, to the delinquency of those whom we would fain defend—or, why the necessity for leaving at the office of each and every one a special notice, together with a copy of the act of assembly for 1819, providing for the registering of births, which we believe has been done?

To our knowledge, there are many in our city who have never registered their names at the Health Office as the law requires, and others who have complained bitterly that the Board of Health require them to furnish their statistics. But why complain? The Board are doing no more than carrying out the designs of a law enacted by the Legislature, at the instance of members of our own profession, and if medical men will not appreciate the subject, and interest themselves in furnishing these statistics, who will do it? We are far from believing that this apparent indifference and dissatisfaction prevail to any extent even among mere routine practitioners, much less with those who, by a studious devotion to their profession, cultivate medicine as a science of the highest order.

We cannot but hope, therefore, that the practitioners of midwifery in this city, will afford every facility within their power, for the collection of the deficient registration of births under their care, for the last three years.

The advantages resulting to the profession and to the community from a well digested and complete system of registration, require no repetition here, inasmuch as the matter has been recently laid before them, and the general public, in a memorial addressed to our State Legislature. The subject in all its important details has also been fully discussed and elucidated, in the councils of our American Medical Association, and by an unanimous vote of that body, an address has been prepared and forwarded to the several state governments, urging upon them, the adoption of measures for carrying out a general registration of births, marriages and deaths.

The present system of registration in Pennsylvania, limited as it is to this city, and full of imperfections, shows interesting results in the detailed investigations furnished at different periods by Dr. G. Emerson, in his tabular statistics; but, if this department of Hygiene was placed on a broader and firmer basis, sanctioned and enforced by legislative enactments, embracing the whole state, we have reason to believe, that, from the accumulation of statistical facts and the deductions made therefrom, that results would accrue, not only for the promotion of the health and longevity of the masses, but for the improvement of the moral and social condition of society, and the well being of the whole Commonwealth.

We have been requested to call the attention of our readers to the advertisment of a physician desirous of disposing of his practice, which will be found in this number; we have been assured, by a reliable person, that it yields two thousand dollars a year.

